# Can healthy ageing moderate the effects of population ageing on economic growth and health spending trends in Mongolia? A modelling study

**DOI:** 10.1186/s12961-022-00916-0

**Published:** 2022-11-29

**Authors:** Gemma A. Williams, Jonathan Cylus, Lynn Al Tayara, Tomáš Roubal, Tsolmongerel Tsilaajav, Sarah L. Barber

**Affiliations:** 1 European Observatory on Health Systems and Policies, London School of Economics and Political Science, Houghton Street, London, WC2A 2AE England; 2 London School of Economics and Political Science, London, England; 3 London School of Hygiene and Tropical Medicine, London, England; 4 Country Office in Ukraine, World Health Organization, Kyiv, Ukraine; 5 Regional Office for South-East Asia, World Health Organization, New Delhi, India; 6 Centre for Health Development, World Health Organization, Kobe, Japan; 7 Office for Health Systems Financing, World Health Organization, Barcelona, Spain

**Keywords:** Population ageing, Healthcare expenditures, Economic growth, Healthy ageing, Mongolia

## Abstract

**Background:**

Population ageing will accelerate rapidly in Mongolia in the coming decades. We explore whether this is likely to have deleterious effects on economic growth and health spending trends and whether any adverse consequences might be moderated by ensuring better health among the older population.

**Methods:**

Fixed-effects models are used to estimate the relationship between the size of the older working-age population (55–69 years) and economic growth from 2020 to 2100 and to simulate how growth is modified by better health among the older working-age population, as measured by a 5% improvement in years lived with disability. We next use 2017 data on per capita health spending by age from the National Health Insurance Fund to project how population ageing will influence public health spending from 2020 to 2060 and how this relationship may change if the older population (≥ 60 years) ages in better or worse health than currently.

**Results:**

The projected increase in the share of the population aged 55–69 years is associated with a 4.1% slowdown in per-person gross domestic product (GDP) growth between 2020 and 2050 and a 5.2% slowdown from 2020 to 2100. However, a 5% reduction in disability rates among the older population offsets these effects and adds around 0.2% to annual per-person GDP growth in 2020, rising to nearly 0.4% per year by 2080. Baseline projections indicate that population ageing will increase public health spending as a share of GDP by 1.35 percentage points from 2020 to 2060; this will occur slowly, adding approximately 0.03 percentage points to the share of GDP annually. Poorer health among the older population (aged ≥ 60 years) would see population ageing add an additional 0.17 percentage points above baseline estimates, but healthy ageing would lower baseline projections by 0.18 percentage points, corresponding to potential savings of just over US$ 46 million per year by 2060.

**Conclusions:**

Good health at older ages could moderate the potentially negative effects of population ageing on economic growth and health spending trends in Mongolia. Continued investment in the health of older people will improve quality of life, while also enhancing the sustainability of public budgets.

## Introduction

Mongolia is in the early stages of a significant demographic transition. Sustained economic and social progress and health system improvements have led to a substantial rise in life expectancy and a rapid decline in the fertility rate during the past three decades [1]. These trends are expected to continue in the future, leading to a considerable and rapid increase in the number and share of older people in the population. Although Mongolia’s population will remain relatively young overall, it is estimated that from 2020 to 2060, the proportion of the population older than 65 years will more than treble, from 4.3% to 14.6%, while the share of people older than 80 years will increase almost fivefold, from 0.63 to 3.1% [1]. This rate of transition is similar to that experienced by Japan six decades before, which saw the share of people aged ≥ 65 years treble, from 5.6% in 1960 to 17.0% in 2000, but it is much faster than that in some other countries in the WHO Western Pacific Region; for example, it took 60 years for the older population in Australia and New Zealand to double from approximately 8.0% in 1960 to 16.0% in 2020 [1].

Population ageing in Mongolia should be viewed as a great achievement. However, the extent and pace of this demographic shift in upcoming decades will have important implications for society, the economy and the health system. The literature highlights two areas of potential concern in particular. First, an important body of research from different settings including the United States [2], Asia [3] and countries in the Organisation for Economic Co-operation and Development (OECD) [4] suggests that having an increasing share of the population that is aged ≥ 55 years may have detrimental effects on economic growth. This is theorized to occur for a number of reasons, including having a smaller share of the population that is of traditional working age and potentially lower productivity rates if older people stay in formal work [5, 6]. Second, data from a wide range of countries at different stages of demographic transition and health system development show a positive association between increasing calendar age and higher healthcare expenditures [7]. This has often led to an assumption that population ageing will contribute to a substantial increase in public health spending, which may prove difficult to manage for some countries. In Mongolia, it is possible that the cost implications of population ageing for health and long-term care may be amplified as the tradition of family members providing support and care for older people is weakened due to smaller family sizes, enhanced labour force participation by women and increased rural–urban and outward migration [8]. These changes are likely to lead to a greater reliance on formal health and long-term care structures in the future.

These two challenges are of course linked, as reduced economic growth and productivity will make it harder to generate revenues to pay for healthcare at a time when an ageing population is increasing the demand for healthcare. However, despite potential concerns over the relationships between population ageing and economic growth and health spending, there is evidence to suggest that these consequences are not inevitable and policy action can be taken to moderate any adverse effects. Evidence has shown, for example, that the relationship between economic growth and population ageing varies depending on the education levels of older workers [9, 10] and whether older workers can use new and emerging technologies that enhance productivity [11, 12]. Meanwhile, the implications for growth in health expenditures depend on a wide range of factors, such as how health services are delivered, the prices paid for goods and services and how coverage decisions are made [7]. Research also indicates that the promotion of healthy and active ageing can reduce the detrimental effects of population ageing on both the economy [13] and health spending [14] by lessening the burden of disease and disability for older people, helping them to remain active and productive for longer and reducing the demand for healthcare [15].

The evidence above suggests that the promotion of healthy ageing is a potentially important policy avenue that can help Mongolia mitigate any adverse consequences of population ageing on its economy and health system. The benefits of healthy ageing have already been recognized by the country’s policy-makers, with the promotion of good health at older ages being an established policy priority. A new national programme (2019–2023) has recently been launched to improve the livelihoods and quality of life of older people (aged ≥ 60 years), which emphasizes ensuring health and healthy ageing, among other goals [16]. The programme follows on from the national programme on healthy ageing and the health of the older persons, which covered the period 2014–2020, and a commitment to protect the health and social welfare of older people has been operationalized in both health and social sector strategies [8]. This focus comes amid a rapidly rising prevalence of noncommunicable diseases and high rates of disability, with 25% of people aged ≥ 60 years in 2016 estimated to be moderately or severely dependent on others to perform instrumental activities of daily living [8]. In the absence of policy action, the burden of noncommunicable diseases is set to continue rising in the coming decades as a result of unhealthy lifestyles, with approximately half of adults being obese or overweight and 43.7% of adult males estimated to be smokers in 2019, according to the fourth national STEPS survey, and high levels of alcohol consumption posing a major public health challenge [17].

While promoting good health and well-being at older ages is a policy priority in Mongolia, the potential benefits of healthy ageing for economic growth and trends in health spending remain unclear. Firstly, few studies have empirically examined how the relationships between population ageing and the economy and health spending may be influenced by better health at older ages. Secondly, as far as we are aware, no studies have considered the effects of healthy ageing on both “where the money for health systems will come from” and “how the money is spent”. Yet, bringing these two elements together is important in order to understand the potential macroeconomic implications of population ageing on the fiscal sustainability of health systems. Overall, a better understanding of the likely implications of healthy ageing will be important for policy-makers to help Mongolia adapt to the challenges of a growing share of older people in the population. This study, therefore, aims to add to the empirical literature and provide evidence for policy-makers by using simulation models to estimate (i) whether having an increasing share of the working population at older ages (55–69 years) is historically associated with a slowdown in real per capita gross domestic product (GDP) growth in Mongolia and to what extent this relationship is moderated by a healthier working-age population, as measured by fewer years lived with disability (YLD); and (ii) whether having an increasing share of the population at older ages (≥ 60 years) will drive an increase in health spending from 2020 to 2060 and whether these effects are reduced by healthy ageing.

The remainder of the paper is structured as follows. First, we review the literature that links changes in population age structure to economic growth and health spending, before providing background information on health spending and the health system in Mongolia. We next outline the data sources and methods used for our analysis. Results from our projections on economic growth for the period 2020–2100 are then provided, followed by estimates on health spending trends for 2015–2060. In our final section, we discuss our findings and their implications for policy.

## Background

### Population ageing and economic growth

The relationship between population age structure and the economy has been widely studied in the academic literature. The vast majority of research finds an inverted U-shaped relationship, in which having a larger share of the population at both younger and older ages is associated with comparatively slower economic growth, whereas having a larger share of the population at middle age is associated with more rapid economic growth. One possible explanation is that both the very young and very old are economically inactive; in the latter case, older people may retire from the formal labour force or otherwise be comparatively less productive than those at younger working ages.

The seminal work by Fair and Dominguez in 1991 is among the first studies to quantify the relationships between age structure and economic variables, using data over more than 30 years from the United States [18]. Their methodology has been replicated and adapted for a wide range of countries at different levels of economic development, with studies generally finding similar results. A 2008 study by Bloom et al. examined the role of demographic change, finding it to be a key factor in East Asia’s economic successes between 1965 and 1990 [3]. The analysis suggested that economic growth has been largely driven by declines in fertility and gains in life expectancy. As a result, the authors predicted that while the early stages of population ageing have been beneficial, future effects, as captured through increases in the old-age dependency ratio, will ultimately lead to slowdowns in growth. Another study in 2012 by Dao considered the links between demographics and economic growth in 43 developing economies, again finding that the old-age dependency ratio had a negative correlation with per capita GDP growth [19]. Likewise, a 2019 study from the Asian Development Bank found that the share of the population aged 10–54 contributes positively to economic growth, with much of the effect concentrated in the group aged 25–34 years [20]. However, increases in the share of the population older than 55 years are correlated with slowdowns in economic growth.

Although the aforementioned study by the Asian Development Bank considers whether technology might mitigate the effects of ageing on growth, few studies have considered whether other factors amenable to policy intervention might moderate the relationship between age structure and economic growth. A 2021 study by Cylus and Al Tayara followed an analogous approach to Fair and Dominguez, but considered whether the health of the older workforce could moderate any effects of a large older workforce [13]. Using data from 1990 to 2017 from 180 countries, the authors found that although a large older workforce (i.e. those aged 55–69) is associated with slower real growth in per capita GDP; however, if that older demographic is in comparatively better health, as measured by YLD, the effects of an ageing workforce on the economy are moderated [13]. This suggests that economic slowdowns attributable to population ageing are not inevitable but, rather, can potentially be avoided through policy intervention.

### Population ageing and health spending trends

As already noted, it is sometimes assumed that having a rising share of older people will drive a substantial increase in health spending due to the higher observed per capita levels of healthcare spending for older people, particularly in more developed health systems. Yet an important body of research suggests that this is not necessarily the case, showing that it is not actually calendar age that is directly linked to higher per-person levels of health spending. Instead, other factors, such as chronic health conditions, dependency or high spending levels at the end of life, are better predictors of health expenditures than calendar age by itself [21–23]. The distinction is important because while population ageing is an unavoidable and indeed welcome consequence of societal progress, these other factors may be amenable to policy intervention.

Most research concludes that population ageing on its own is unlikely to be a major driver of increases in health spending. Research exploring the drivers of past growth in health spending seemingly confirms this hypothesis. For example, in a 2013 study, Medeiros and Schwierz determined that from 1985 to 2010 only 7% of the increase in public health expenditure in the European Union and Norway was driven by demographic factors [24]. Similarly, de la Maisonneuve and Oliveira Martins in 2013 found that public health spending in OECD countries grew by an average of 4.3% per year between 1995 and 2009, with only 0.5% attributed to population ageing [25]. Comparable results were found for the United States, with an analysis of five household surveys revealing that between 1970 and 2000, only 0.2 percentage points of the 4.3% annual growth rate of Medicare expenditures were linked to population ageing [26].

Research projecting the impact of demographic changes on future health expenditures indicates that this situation is unlikely to change, with population ageing remaining a minor driver of health spending growth in the coming decades. Nevertheless, it should be noted that projections vary by country, reflecting the pace of population ageing, levels of GDP, provision of publicly funded long-term care and how older patients are managed in the health system. Projections from the European Commission, for instance, forecast that demographic changes will bring about increases in public expenditure on healthcare in Member States by 1.9% of GDP, on average, between 2007 and 2060 [27]. The impact of population ageing on growth in public health expenditures will vary between Member States, ranging from 0.7 to 3.8 percentage points, with the largest impact seen in recently acceded countries from Eastern Europe [27]. OECD projections report similar findings, with estimates finding that, on average, demographic change could bring about increases in spending on healthcare from 5.5% of GDP in 2010 to 6.2% in 2060 in OECD countries, and from 2.4% of GDP in 2010 to 3.4% in 2060 in non-OECD countries [25]. This translates to a relatively small addition to GDP, with estimates varying from 0.2 percentage points of GDP in Belgium, Iceland and the United Kingdom to more than 1.8 percentage points in Chile, the Republic of Korea and Turkey [25].

While many studies have investigated the impact of changes in the population age structure on the growth in health spending, few have explored how policy actions to promote healthy ageing and reduce dependency at older ages might alter this relationship. In a notable exception, Nozaki and colleagues conduct a decomposition analysis to determine the likely impact of population ageing on growth in healthcare and long-term care spending in Japan and how these estimates would be affected if the future health status of older people improved [14]. The study determined that population ageing by itself, in the absence of any policy changes, would increase spending on health and long-term care by 3.4 percentage points—from 9.5% to 13% of GDP—between 2010 and 2030. However, introducing a hypothetical improvement in health status for older people along with a continued increase in life expectancy would reduce this estimated increase by 1 percentage point of GDP across the same period [14]. Therefore, this provides some evidence that taking steps to promote healthy ageing and reduce dependency at older ages may help moderate any projected increase in health spending as a consequence of population ageing.

### Mongolia’s health system

Mongolia’s health system is primarily funded by general taxation and social health insurance [28]. Spending as a share of GDP increased slightly from 3.8 to 4.0% from 2005 to 2017, with per capita health spending more than doubling from US$ 74 to US$ 149 over the same period [29]. Tax-financed government expenditure on health accounted for 41.0% of current health expenditure in 2017, with social health insurance 23.0% of current health expenditure [29]. Out-of-pocket payments accounted for approximately one third of total health spending in 2017 as a result of user charges and cost-sharing for outpatient and inpatient care. Government expenditure covers the provision of an essential package of services in primary healthcare and some secondary and tertiary level services. Social health insurance covers most outpatient services, major inpatient services and a proportion of the cost of medicines, day care, home care, rehabilitation and certain diagnostic tests [30].

## Methods

### Data sources

For all projections, forecasted data on population by age from 2020 to 2100 were obtained from the United Nations’ World Population Prospects 2019 [1].

For our first set of projections, exploring the relationship between a higher proportion of the population at older ages and economic growth, we used age-specific YLDs as a measure of health and disability, with data taken from the Institute for Health Metrics and Evaluation [31]. We opted to use YLDs rather than disability-adjusted life-years (DALYs) because DALYs capture premature deaths and not just disability rates. To calculate GDP growth rates, we used real (price inflation-adjusted) per capita GDP measures in local currency units obtained from World Bank World Development Indicators.

**Fig. 1:**
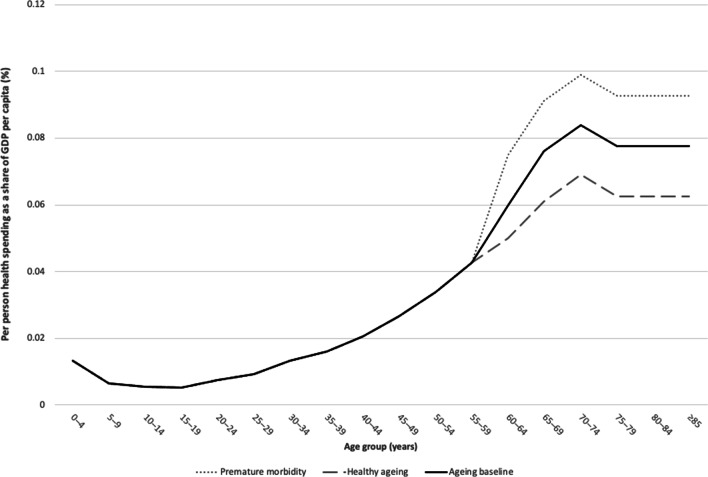
Per-person social health insurance expenditure as share of gross domestic product (GDP), by age group (baseline and two hypothetical scenarios), 2017, Mongolia. Figure created by the authors from data in [27]

For our second set of projections, exploring the influence of population ageing on growth in health spending, we took data on per capita public health spending by age in 2017 from an actuarial review of the National Health Insurance Fund (NHIF) of Mongolia (WHO, unpublished data from actuarial review of the NHIF, 2019) (Fig. 1). Baseline data included health spending for services covered by the benefit package of the NHIF, which includes inpatient services, outpatient care, some rehabilitative care, home care, day care, diagnostic tests provided by local health centres, palliative care, haemodialysis and various high-cost procedures, such as chemotherapy and radiotherapy for cancer treatment. It should be noted that these baseline data include only one third of public health spending, but they were the only data available for our estimates. The data nevertheless provide an understanding of how public health spending is distributed by age, which we assume applies to total health spending. Moreover, it compares reasonably well with data from other countries with more complete data that are likely to have a similar spending by age profile, such as Vietnam (see for example [32]).

As shown in Fig. 1, expenditure for the NHIF by age group follows a similar pattern to that seen in other countries, with per capita health spending as a share of GDP comparatively high at birth until 1 year of age and then remaining relatively low until about 50 years of age. At this age, health expenditures start to steadily increase until 70 years of age, before declining for those older than 75 years.

### Empirical strategy

For the first set of analyses, we followed the empirical strategy proposed by Cylus and Al Tayara and use fixed-effects models to estimate the historical relationship between population ageing and economic growth between 1990 and 2017 and consider the potential moderating effects of better health and reduced disability among the older working-age population; we then use these models to forecast the future effects of an ageing workforce on economic growth [13]. We produce estimates for Mongolia and a selection of countries (Australia, Japan, New Zealand, the Republic of Korea and Viet Nam) from the Western Pacific Region as we aim in subsequent models to compare effects across countries at different stages of the demographic transition and with different levels of health system development.

The baseline model is specified in (1) below:

1 $$\begin{aligned} {\text{lnGDPcapit}} - {\text{lnGDPcapit}} - 1 = & {\text{c}} + \beta {\text{1PopShare}}\left( {20{-}39} \right)_{{{\text{it}}}} + \beta {\text{2PopShare}}\left( {40{-}54} \right)_{{{\text{it}}}} \\ & + \beta {\text{3PopShare}}\left( {55{-}69} \right)_{{{\text{it}}}} + {\text{C}}_{{\text{i}}} + {\text{T}}_{{\text{t}}} + {\text{u}}_{{{\text{i,t}}}} \\ \end{aligned}$$

In model (1), log-differenced real per capita GDP in country i and year t is modelled as a function of population age shares in country i and t. Fixed effects for country and year are included to control for time-invariant country characteristics and to capture developments over time that affect the entire sample of countries.

We next model annual, real per capita GDP growth as a function of the share of the predicted population aged 55–69 years (specified in (2) below). We include only the population share aged 55–69 years as an explanatory variable in the model as opposed to a range of population age groups because population age shares are highly collinear.

2 $${{{\text{lnGDPcap}}}}_{{{\text{i}},{\text{t}}}} {-} {{{\text{lnGDPcap}}}}_{{{\text{i}},{\text{t}} - {1}}} = {\beta }_{{1}}{{{\text{PopShare}}({55} - {69})}}_{{{\text{i}},{\text{t}}}} +{{\text{C}}}_{{\text{i}}} +{{\text{T}}}_{{\text{t}}} +{{\text{u}}}_{{{\text{i}},{\text{t}}}}$$

In step 3, the main effect of inverse YLDs and the interaction between YLDs and share of workers in the 55–69 age group are incorporated to test whether the potential adverse effects of an older working-age population share were moderated by better health and disability levels in that age group [model (3)].

3 $${{{\text{lnGDPcap}}}}_{{{\text{i}},{\text{t}}}} {-} {{{\text{lnGDPcap}}}}_{{{\text{i}},{\text{t}} - {1}}} = {\beta }_{{1}}{{{\text{PopShare}}({55} - {69})}}_{{{\text{i}},{\text{t}}}} +{\beta }_{{2}}{{({1}/({\text{YLD5569}}}}_{{{\text{i}},{\text{t}}}}{)) + \beta }_{{3}}{{({1}/{\text{YLD5569}}}}_{{{\text{i}},{\text{t}}}} *{{{\text{PopShare}}({55} - {69})}}_{{{\text{i}},{\text{t}}}}{)} +{{{\text{lnGDPcap}}}}_{{{\text{i}},{\text{t}} - {2}}} {{\text{C}}}_{{\text{i}}} +{{\text{T}}}_{{\text{t}}} +{{\text{u}}}_{{{\text{i}},{\text{t}}}}$$

In model (3) the log of real per capita GDP (at t-2 for the annual growth models, and at t-3 for the 5-year centred average) are included to account for country-specific time varying characteristics (e.g. technological innovation, education, or unemployment rates) that correlate with the level of GDP per capita. Inverse YLDs are preferred because larger values indicate better health and, thus, coefficients are easier to interpret. To estimate the magnitude of the effects of supporting healthy ageing in Mongolia, we used these models to forecast the per-person growth in GDP while holding constant baseline per capita 2017 YLDs for the 55–69 year age group and compared these projections to a second scenario that assumed disability rates are held constant, but at 5% lower than baseline. The 5% value was chosen to estimate how even a small improvement in health might have benefits for economic growth.

Our second set of analyses estimates the relationship between having an increasing proportion of the population in older age groups in Mongolia and growth in public health spending, and how these estimates are influenced by older people ageing in worse or better health than currently.

In our baseline model, we multiplied per capita public health spending from 2017 NHIF baseline data by the size of the population in each respective age group from 2015 to 2060. The resulting total expenditure across all age groups was then divided by the total population size to give us a measure of per capita health spending that varies from year to year only as a result of changes to the population age structure [health spending model (1)]. This approach assumed that patterns of health spending by age remain constant and, thus, any changes in expenditure levels as a results of prices, technologies or a change in entitlements have similar effects across age groups. In other words, if at baseline healthcare spending for an 80-year-old is five times that for a 20-year-old, then this distribution of spending remains constant over time even if total spending increases.

This baseline model was then adjusted to assume that people age in worse health [heath spending model (2)] or better health [health spending model (3)] than currently. In health spending model (2), we assumed that older people age in worse health than currently, a scenario we refer to as premature morbidity, leading to higher health spending for older age groups compared with baseline levels. To simulate this scenario, baseline per-person health expenditures in Mongolia were adjusted so that health spending for the 60–64 and 65–69 years age groups is 1.00 percentage point higher than currently and 1.50 percentage points higher for age groups ≥ 70 years (Fig. 1). In our final model [health spending model (3)], we simulated the effects of healthy ageing, which is assumed to lead to lower healthcare costs for older age groups compared with baseline levels. To do so, we adjusted baseline per capita health spending so that health spending for each age group from 60 to 69 years is 1.00 percentage point lower than currently and 1.50 percentage points lower for age groups ≥ 70 years (Fig. 1). Using this adjusted data on baseline health spending, we then used the approach outlined above of multiplying per capita health spending by age group by respective age group populations in each year, before dividing the total estimated spending in each year by the projected population size.

## Results

In this section, we first present results on the relationship between population ageing in Mongolia and economic growth, before looking at the association with trends in healthcare expenditure.

### Population ageing and economic growth

Results from economic growth model 2, which does not adjust for health and disability, indicated that an increase in the share of population aged 55–69 years has a negative and statistically significant association with real GDP growth. According to model estimates, the projected increase in the share of the population aged 55–69 from 9.27 to 15.28% between 2020 and 2050 in Mongolia is expected to contribute to a slowdown in per-person GDP growth of around 4.1% overall. In longer projections from 2020 to 2100, an increase in the share of the population aged 55–69 from 9.27% to 16.86% is expected to reduce per-person GDP by around 5.2% overall.

**Fig. 2:**
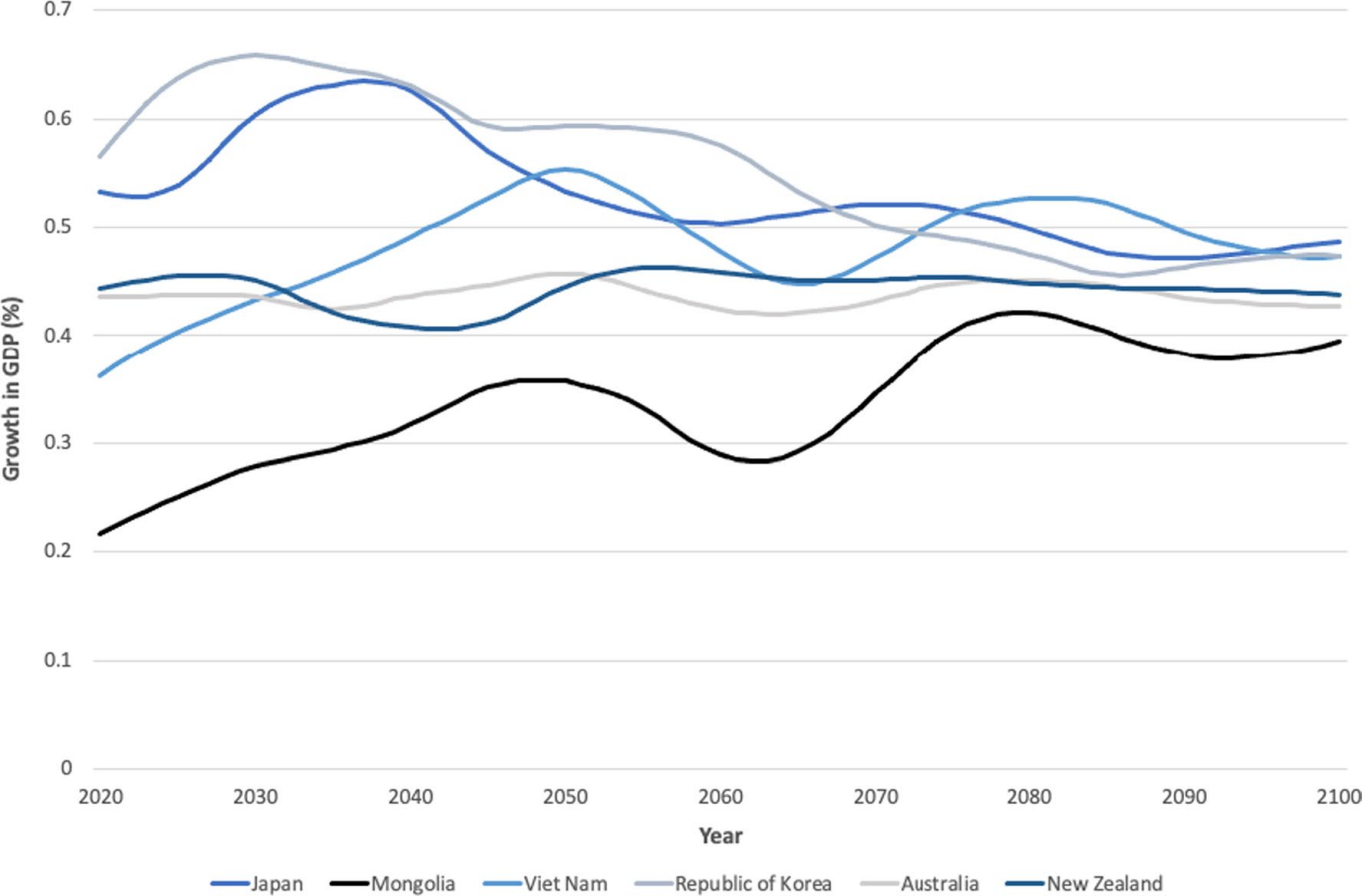
Growth in gross domestic product (GDP) 2020–2100 attributable to a 5% improvement in disability rates among population aged 55–69 years compared with 2017 baseline rates. Source: Authors’ calculations

In economic growth model 3, we accounted for the level of health and disability among those aged 55–69 years by comparing two scenarios in which inverse baseline YLDs for this age group were held constant over the projection period to a second scenario in which YLDs were held constant but at 5% lower than baseline. Model estimates suggested that better health and less disability among this age group can help moderate any adverse effects of population ageing and contribute positively to GDP growth. Figure 2 shows that a 5% reduction in disability rates among the population aged 55–69 years in Mongolia adds approximately 0.2% to annual per-person GDP growth in 2020, rising to about 0.4% in 2080, thus more than offsetting the effects of an ageing workforce. In all other countries, a 5% reduction in disability rates among the population aged 55–69 years similarly leads to a positive contribution to economic growth. This contribution is highest in Japan and the Republic of Korea in 2020, adding approximately 0.55% to annual per-person GDP growth. Unlike for Mongolia, however, the contribution to GDP growth declines in both countries due to changes in the age-mix of the population to just under 0.5% in 2100, although it remains positive. The differing trends in Mongolia compared to Japan and Republic of Korea reflect the different stages of the demographic transition that these countries have already reached and will undergo over the 80 next years; in other words, while Mongolia’s population is comparatively younger now, the share of the population in older age groups will increase more rapidly over the coming decades.

**Fig. 3:**
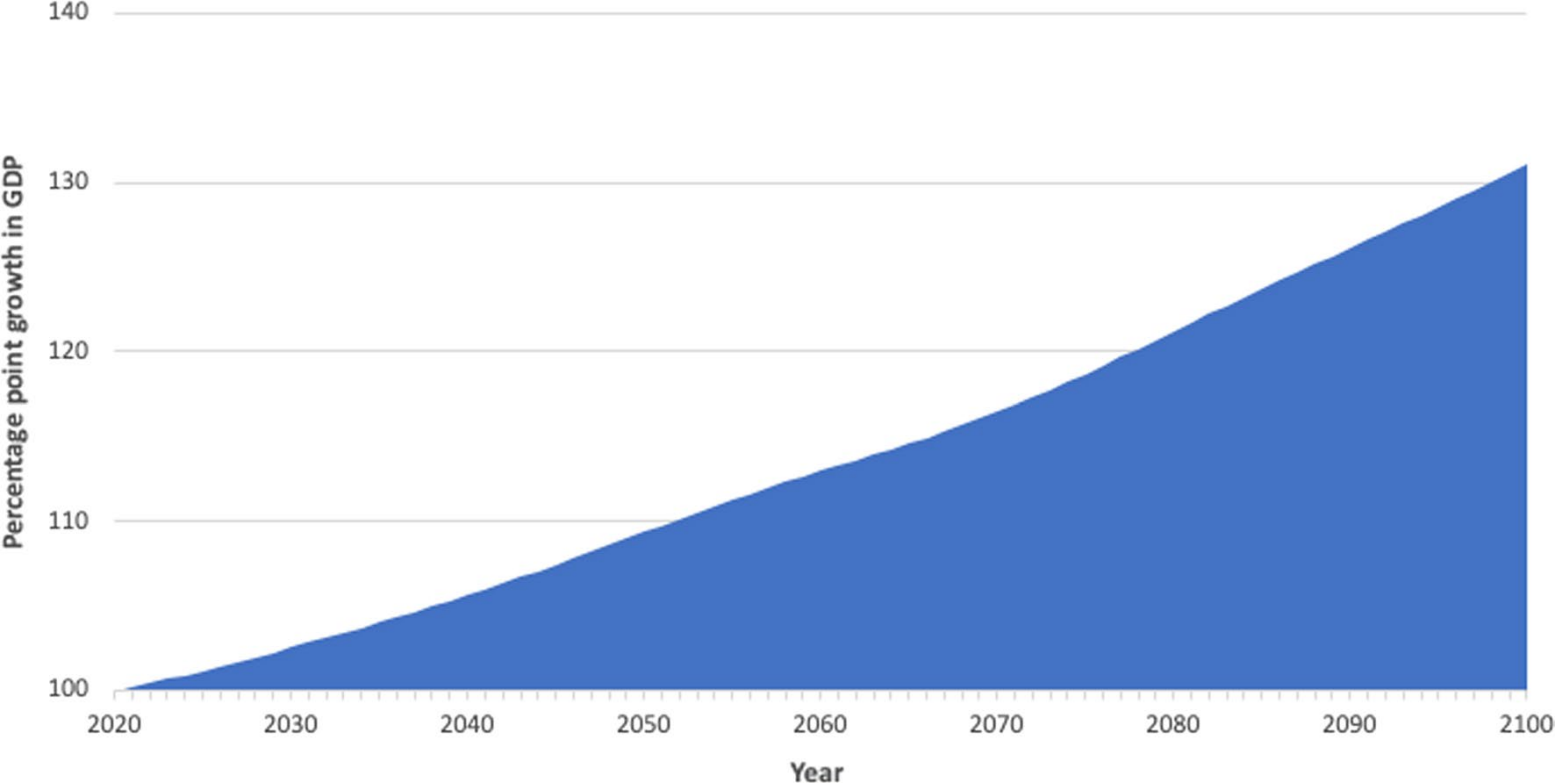
Cumulative growth in gross domestic product (GDP) attributable to a 5% improvement in disability rates among the population aged 55–69 years compared with 2017 baseline rates (index year is 2020 = 100 points). Source: Authors’ calculations

From 2020 to 2100, estimates indicate that Mongolia could expect to see an additional 31.1 percentage points of GDP growth per person in total if disability rates among those aged 55–69 years were constant but 5% lower than in 2017 (Fig. 3).

### Population ageing and health spending trends

**Fig. 4:**
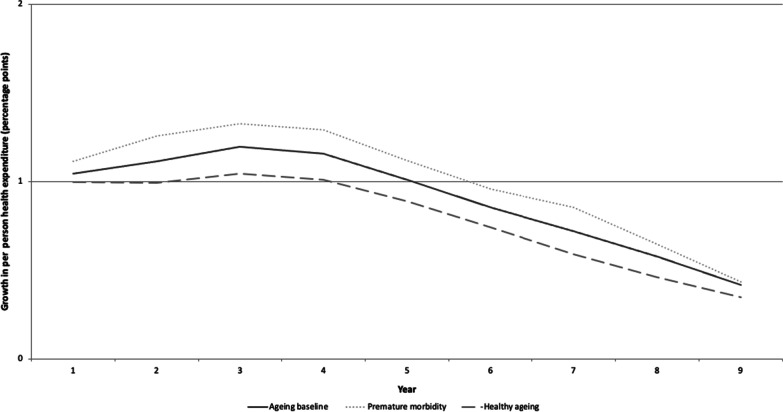
Projected additional growth in per-person public health expenditure (percentage points) attributable to population ageing, Mongolia, 2015–2060. Source: Authors’ calculations

In health spending model 1, we projected growth in health spending in Mongolia using 2017 baseline data on per capita health spending as a share of GDP by age group (Fig. 1). The model estimates suggested that growth in public health expenditure due to population ageing is expected to be relatively low through 2060—with population ageing contributing 1.19 additional percentage points per year to the growth in average annual per-person healthcare spending between 2025 and 2030—before this contribution declines to 0.41 percentage points per year by 2060 (Fig. 4). As a comparison, the average nominal growth rate of per-person total health spending due to all causes in Mongolia between 2013 and 2017 was approximately 9.9%. If this trend continued to 2060, factors other than population ageing, notably prices, volume of care and technology adoption, would therefore account for the majority of growth in health spending in Mongolia.

**Fig. 5:**
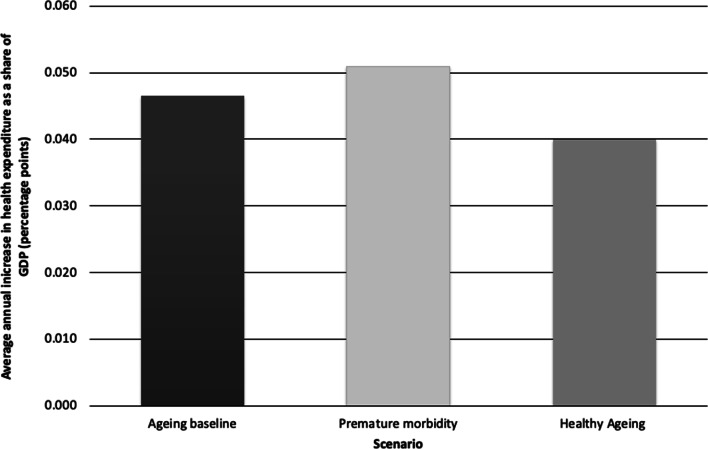
Average annual increase in public health expenditures as a share of gross domestic product (GDP), Mongolia, 2020–2060, resulting from population ageing under three scenarios: baseline health expenditure by age, healthy ageing and premature morbidity. Source: Authors’ calculations

These estimates indicate that population ageing will increase public health spending as a share of GDP by 1.35 percentage points from 2020 to 2060. This is not an insignificant share of the economy, but this increase will occur slowly, with the average increase over the period being slightly more than 0.03 percentage points annually (Fig. 5).

We then adjusted health spending model 1 to assume that people ≥ 60 years age in worse health than they do currently and, thus, account for higher healthcare expenditures than in the baseline NHIF data from 2017. Under this premature morbidity scenario, the additional growth in average annual spending per person attributable to population ageing would peak between 2025 and 2030 at 1.33 percentage points per year before steadily declining to 0.44 percentage points per year in 2060 (Fig. 4). Over the same projection period, population ageing that assumed premature morbidity would increase public health expenditures as a share of GDP by 1.53 percentage points, an increase of 0.17 percentage points above the estimates in health spending model 1, which used baseline health expenditure data. Projections from health spending model 2 indicated that the average increase in the share of public spending on health as a result of population ageing would be just under 0.04 percentage points per year during the 40-year period (Fig. 5).

In our final model, we simulated the effects of healthy ageing by assuming that people ≥ 60 years age in better health than they do currently and, thus, have lower health spending costs than indicated by baseline data. Estimates from this hypothetical scenario indicate that the additional growth in average annual public health spending per person that is attributable to population ageing would peak at 1.05 percentage points per year between 2025 and 2030 before declining to 0.35 percentage points per year in 2060 (Fig. 4). In this hypothetical healthy ageing scenario, public health expenditure as a share of GDP would increase by 1.18 percentage points between 2020 and 2060 as a result of population ageing. This is 0.18 percentage points lower than estimates from health spending model 1, which used baseline health expenditures. The average increase in the share of the economy spent on health as a result of population ageing under a healthy ageing scenario during the 40-year period would be just under 0.03 percentage points per year (Fig. 5).

A comparison of our two hypothetical scenarios suggests that if people age in good health public health spending would consume 0.35 fewer percentage points of GDP by 2060 than if people age in poor health. In monetary terms, this corresponds to just under 0.01% of GDP per year during the next 40 years. Based on 2018 GDP estimates, this would amount to savings in public spending on health of almost US$ 46 million per year by 2060. However, we should emphasize that these figures are purely illustrative and based on hypothetical scenarios and should not be viewed as forecasts of savings in future health spending.

## Discussion

Population ageing is occurring at a rapid pace in Mongolia. However, in contrast to some fears, results from our analysis suggest that a growing number and share of older people in the population will not inevitably pose insurmountable challenges to the health system and the economy. Moreover, any potentially adverse effects of population ageing on economic growth and health spending can be moderated by promoting better health at older ages.

Our first set of simulations looking at economic growth in Mongolia suggest that an increase in the share of the population aged 55–69 is expected to coincide with a slowdown of per-person GDP growth of around 4.1% between 2020 and 2050 and by 5.2% in longer-term projections, from 2020 to 2100. The negative correlation between GDP growth and population ageing confirms findings from previous studies from East Asia [3] and developing countries more widely [19].

This is concerning, as it is possible that any slowdown in economic growth and public revenues may lead to lower investments in health and other social sectors in an attempt to contain costs and reduce public budgets. However, we find that this slowdown can be offset by relatively small improvements in the rates of disability among older people. A 5% improvement in disability rates among those aged 55–69 years could add an additional 31.1 percentage points of GDP growth per person by the end of the century. These findings echo those from Cylus and Al Tayara in 2021, which showed that better health among the older workforce could help moderate any adverse effects of population ageing on economic growth [13]. This suggests that countries like Mongolia, which are at a relatively early stage of the demographic transition, should make use of this position and promote healthy and active ageing now among the working-age population to reap these potential economic benefits.

We also found that ensuring better health at older ages is likely to have tangible benefits for trends in health spending. Our initial projections suggest that population ageing in the absence of policy changes will increase public health expenditures as a share of GDP by 1.35 percentage points between 2020 and 2060. This will place pressure on public health budgets, although it is important to note that this increase will occur slowly, at an average of just more than 0.03 percentage points per year. Moreover, as already highlighted, If past trends continue, this means population ageing will account for a small share of growth in health spending compared to other factors such as prices, volume of care and technology adoption. This is consistent with other studies that have determined calendar ageing by itself is not a primary predictor of health spending compared to other factors often linked to age such as chronic health conditions, dependency or high spending levels at the end of life [21–23].

Importantly, we show that if per capita levels of health spending for older age groups were to increase in the future as a result of poorer health and higher rates of disability, the impact of population ageing on public health expenditures may be greater than anticipated (by an additional 0.17 percentage points of GDP over the 40-year period), but working towards better health at older ages was found to reduce the growth in public health spending to around 1.18 percentage points during the same period. Of course, achieving a 5% reduction in disability may require additional health spending on better public health programmes and improved access to healthcare, which may reduce potential savings. Nevertheless, many of the most successful “best buy” preventative measures such as taxes, bans on advertising or mass media campaigns against smoking, excessive alcohol use or unhealthy foods have been found to be cost-effective interventions that are exogenous to the health budget [33]. It is therefore possible that a reduction in the disability rate may be achieved without excessive additional costs for the health sector.

## Limitations

Our study has several limitations beyond those already noted in our Methods section. In our models on economic growth, we are unable to unequivocally demonstrate that health has a causal effect on economic growth. We are also unable to capture the effects of changes in the nature of work in future (e.g. as the result of the introduction of a new technology) that may influence productivity rates and labour market decisions. To isolate the impact of population ageing on health spending from other factors, we assumed that baseline heath expenditures by age remain constant across the entire projection period. However, this profile of baseline spending by age could differ in the future as a result of changes in prices, the volume of care delivered and the types of care delivered [7]. In addition, our analysis was also undertaken based on age groups and not at the individual level, which may result in some information loss because this analysis cannot take account of micro-level differences in developments in health and health spending. Finally, our baseline data on health spending do not capture all spending on public health and long-term care, which means our projections carry an element of uncertainty and may be lower than in actuality. Nevertheless, these were the only data available for this research. More routine data collection and surveillance that captures spending on health and long-term care by age in Mongolia could help guide the development of more accurate projections of the potential implications of population ageing for the economy and health system financing.

## Conclusions

While having an increasing share of older people in a population will pose some challenges for the health system and economy in Mongolia, these are likely to be manageable, especially if the population ages in good health. Mongolia already has ambitious plans in place to improve the health and well-being of and employment opportunities for older people, as evidenced by the development of cross-sectoral strategies such as the National Program on the Development and Protection of Elderly People [16] and the National Program on Healthy Aging and Health of Older Persons (2014–2020) [34]. Ensuring effective implementation of these strategies and programmes across all of the WHO regions, while continuing to strengthen primary healthcare and prevention strategies and increase population coverage, will not only help individuals to enjoy better health and longer lives but also can support society’s prosperity and the sustainability of public budgets.

## Data Availability

The majority of data used in this study are publicly available, with data on health spending by age obtained from the National Health Insurance Fund of Mongolia.

## Abbreviations

DALYs: Disability-adjusted life-years
GDP: Gross domestic product
NHIF: National Health Insurance Fund
OECD: Organisation for Economic Co-operation and Development
YLD: Years lived with disability
